# Protein-protein interface hot spots prediction based on a hybrid feature selection strategy

**DOI:** 10.1186/s12859-018-2009-5

**Published:** 2018-01-15

**Authors:** Yanhua Qiao, Yi Xiong, Hongyun Gao, Xiaolei Zhu, Peng Chen

**Affiliations:** 1 School of Life Sciences, Anhui University, Hefei, Anhui 230601 China; 2 State Key Laboratory of Microbial Metabolism, Shanghai JiaoTong University, Shanghai, 200240 China; 3 School of Life Sciences and Biotechnology, Shanghai JiaoTong University, Shanghai, 200240 China; 4 Information and Engineering College, Dalian University, Dalian, Liaoning 116622 China; 5 Institute of Health Sciences, Anhui University, Hefei, Anhui 230601 China

**Keywords:** Protein-protein Interface, Hot spot, Feature selection, Residue conservation

## Abstract

**Background:**

Hot spots are interface residues that contribute most binding affinity to protein-protein interaction. A compact and relevant feature subset is important for building machine learning methods to predict hot spots on protein-protein interfaces. Although different methods have been used to detect the relevant feature subset from a variety of features related to interface residues, it is still a challenge to detect the optimal feature subset for building the final model.

**Results:**

In this study, three different feature selection methods were compared to propose a new hybrid feature selection strategy. This new strategy was proved to effectively reduce the feature space when we were building the prediction models for identifying hotspot residues. It was tested on eighty-two features, both conventional and newly proposed. According to the strategy, combining the feature subsets selected by decision tree and mRMR (maximum Relevance Minimum Redundancy) individually, we were able to build a model with 6 features by using a PSFS (Pseudo Sequential Forward Selection) process. Compared with other state-of-art methods for the independent test set, our model had shown better or comparable predictive performances (with F-measure 0.622 and recall 0.821). Analysis of the 6 features confirmed that our newly proposed feature *CNSV*_*REL*1 was important for our model. The analysis also showed that the complementarity between features should be considered as an important aspect when conducting the feature selection.

**Conclusion:**

In this study, most important of all, a new strategy for feature selection was proposed and proved to be effective in selecting the optimal feature subset for building prediction models, which can be used to predict hot spot residues on protein-protein interfaces. Moreover, two aspects, the generalization of the single feature and the complementarity between features, were proved to be of great importance and should be considered in feature selection methods. Finally, our newly proposed feature *CNSV*_*REL*1 had been proved an alternative and effective feature in predicting hot spots by our study. Our model is available for users through a webserver: http://zhulab.ahu.edu.cn/iPPHOT/.

**Electronic supplementary material:**

The online version of this article (10.1186/s12859-018-2009-5) contains supplementary material, which is available to authorized users.

## Background

Proteins play pivotal roles in almost all biological processes. They do not act as isolated units; instead, they often perform their functions by forming protein-protein complexes. Protein-protein interaction is the foundation for many different cellular processes, such as signal transduction, cellular motion, and regulatory mechanisms [1, 2]. More significantly, proteins are often components of a large protein-protein interaction(PPI)networks, so that the erroneous or disrupted protein-protein interaction can cause diseases [3]. Different aspects of protein-protein interfaces (such as sequences and structures) have been analyzed to explore the rules governing the interaction [1, 2, 4–9]; however, to our best knowledge, the general rules to characterize the interfaces have not been fully extrapolated yet due to the intrinsic complexity of the interfaces.

It implies vital clues for understanding protein-protein interactions to identify residues that are energetically more important for the binding, and it has been proved that the contributions of interface residues to binding are not homogeneous. Actually, the majority of the binding energy can be accounted for by a small part of the interface residues, which are so called hot-spots (HS) [10–12]. The definition of HS is based on alanine scanning mutagenesis [13]: an interface residue is mutated to alanine, and the binding free energy difference (∆∆*G*_*binding*_) between forming the wild type and the mutant complex is calculated. If the ∆∆*G*_*binding*_ ≥ 2.0*kcal*/*mol*, the residue is defined as HS, otherwise the residue is defined as non hot-spot (NS).

Different methods, both experimental and computational, have been developed for identifying HS. As an extensively used experimental method, alanine scanning mutagenesis [13] can detect HS directly and effectively; however, the experimental methods are not only expensive but also time-consuming. Instead, computational methods [14–26] predict HS in silico with higher efficiency and lower cost. Computational methods are generally based on empirical function or on knowledge. Knowledge-based methods, especially the machine learning based methods [21, 24–26], can predict HS with a wide range of features of residues and are more effective than those fully atomic models [14, 15].

Many different kinds of features of interface residues have been used to build machine learning models [21, 24–26], such as residue index, solvent accessible surface area, residue conservation, atom density, and so on. The conservation of residues has been used as a feature in several published methods [19, 24, 26–28], but its effect remains controversial.

Different kinds of features proposed make a large feature space for building models to differentiate HS from NS. Feature selection becomes a key step in building an effective classification model. Feature selection is an important topic in data mining, especially in high-dimensional applications, by which a compact and effective feature subspace can be determined. So that we can avoid over-fitting, improve model performance and provide faster and more cost-effective models. There are mainly two kinds of features selection algorithms: the filter approach and the wrapper approach [29]. The filter approach first selects informative features, then builds the models by classification algorithm. The wrapper approaches either modify classification algorithm to choose important features as well as conduct training/testing or combine classification algorithm with other optimization tools to perform feature selection. Different kinds of feature selection methods have already been used to build machine learning models for predicting HS, such as mRMR [30], decision tree [31], F-score [32] and so on. However, it is still challenging to select an optimal feature subset to construct the classification models. The filter approaches often use a specific metric, such as mutual information, to rank all the features. Although computationally efficient, the filter approaches often do not fully consider the dependences, such as redundancy and complementarity, between features. For example, in APIS [24], Xia et al. used F-score to select relevant features, by which their models were based on single features. While the wrapper approaches easily select a subset of features that overestimate the correlation between features and the labels, which makes the final model over-fitted. For example, in MINERVA [21], Cho et al. used decision tree to select features, by which their model used 12 features that might over-fit their model.

In this work, we proposed a hybrid feature selection strategy by combining three different kinds of feature selection methods. According to the strategy, by combining both filter and wrapper approaches, we were able to build a new model to predict HS on protein-protein interface based on the 6 features selected from 82 features.

## Methods

### Data sets

#### Training data set

Our training data set is the same as that used by Xia et al. [24]. The data set includes 154 interface residues with observed alanine scanning energy differences, categorized into hot spots (HS) and non-hot spots (NS). In this study, the interface residues are defined as those residues whose buried solvent accessible surface areas are larger than 0.0 Å^2^ when binding. The original data were obtained from ASEdb [33] and the published data of Kortemme and Baker [14]. The redundancy of the training data was calibrated by eliminating the protein chains with the sequence identity cutoff 35% and the SSAP (Secondary Structure Alignment Program) score cutoff 80 using the CATH query system. Because we intended to build our models by considering the evolutionary conservation score related features as part of the features, those protein chains that do not have final searching result on the ConSurf server [34] were also removed. By these processes, we obtained 15 protein complexes that contain interface residues with alanine scanning data. The 15 protein complexes are listed in the Table 1. An interface residue is defined as hotspot residue if its mutation to alanine produces a ∆∆*G* ≥ 2.0 *kcal mol*^−1^. An interface residue is defined as non hot spot residue if its mutation to alanine produces a ∆∆*G* < 0.4 *kcal mol*^−1^, as described by Tuncbag et al. [19]. Tuncbag et al. selected these two cutoff values based on the distributions of alanine scanning data for both interface residues and other surface residues as Gao et al. have done in their paper [35]. In the meantime, the remaining interface residues whose binding free energy differences (∆∆*G*) are between 0.4 kcal/mol and 2.0 kcal/mol were removed from our final training data. According to the definition, we obtained 154 interface residues that comprised 62 hot-spot residues and 92 non-hot spot residues, as listed in the Additional file 1: Table S1. The training data set is used both for cross-validation and training the different models.

**Table 1:** The 15 complexes used in the training data set

PDB	First molecule	Second molecule
1a4y	Angiogenin	Ribonuclease Inhibitor
1a22	Human growth hormone	Human growth honnone binding protein
1ahw	Immunoglobulin Fab 5G9	Tissue factor
1brs	Bamase	Barstar
1bxi	Colicin E9 Immunity Im9	Colicin E9 DNase
1cbw	BPTI Trypsin inhibitor	Chymotrypsin
1dan	Blood coagulation factor VI1A	Tissue factor
1dvf	Idiotopic antibody FV D1.3	Anti-idiotopic antibody FV E5.2
1 fc2	Fc fragment	Fragment B of protein A
1fcc	Fc (IGG1)	Protein G
1gc1	Envelope protein GP120	CD4
1jrh	Antibody A6	Interferon-gamma receptor
1vfb	Mouse monoclonal antibody D1.3	Hen egg lysozyme
2ptc	BPTI	Trypsin
3hfm	Hen Egg Lysozyme	lg FAB fragment HyHEL-10

#### Medium test set

Although the interface residues whose mutation to alanine produce ∆∆*G* between 0.4 kcal/mol and 2.0 kcal/mol have been removed from the training data set. We kept them as a medium test set. The test set contains 98 residues with observed ∆∆*G* (Additional file 1: Table S2). This data set is used only for testing the performance of different models.

#### Independent test set

To evaluate and compare the performance of our model and other hot spots prediction methods, an independent test set was derived from the BID database [36]. By being manually checked in the SCOP database [37] in which the protein families are defined by using sequence identity 30%, the proteins in the independent test set are non-homologous to the ones in the training set. If homologous pairs are included, the recognition sites differ between the two proteins. In the BID database, the relative effects of alanine mutation are denoted by “strong”, “intermediate”, “weak” and “insignificant”. In our study, the residues marked “strong” were selected as hot spot residues, and the other residues were considered as non-hot spot residues. In addition, the protein chains that do not have final searching result on the ConSurf server were excluded from our research. Finally, we obtained 95 residues from 16 complexes, 28 of which were hot spot residues and 67 of which were non-hot spot residues (See Additional file 1: Table S3). The independent test set is used only for testing the performance of different models.

### Features representation

In order to predict hot spot residues effectively, we generated a total of 82 features including sequence-based or structure-based features to test feature selection methods and build our model. These features contain 10 physicochemical properties of 20 types of standard amino acids, B factor, 36 features of the structural information of our selected proteins in the unbound and bound states, 5 features related to the evolutional conservation of residues and 30 features related to solvent accessible surface area differences between the unbound and bound states. All features are listed in Additional file 2: Table S4. Note that the first 47 features and the 78th feature in the table have been used in Xia et al.’s work [24]. These features include the ten features of physicochemical properties of 20 amino acids, B factor, the 36 features calculated with PSAIA program [38], and the conservation score generated by ConSurf [34]. The remaining 34 features are new features proposed in this study.

#### Ten features of physicochemical properties of 20 amino acids

The physicochemical properties of residues determine its interactions with the others. Several studies [32, 39–41] have indicated that 10 physicochemical properties were closely related to the interface properties of proteins. These 10 properties consisted of the number of atoms, the number of electrostatic charge, the number of potential hydrogen bonds, hydrophobicity, hydrophilicity, propensity, isoelectric points, mass, the expected number of contacts within 14 Å sphere and electron-ion interaction potential. The 10 properties were used as features in this study. The property values are only associated with the types of amino acid residues, and not allied to any structural information. The numerical values of the 10 features are showed in (See Additional file 2: Table S5).

#### B factor (temperature factor)

B factor is a measure of flexible activities in proteins, which reveals the mobility of the crystalline state of atoms. In previous studies [8], it was demonstrated that the interface residues of proteins were inclined to be rigidity (that is inferiorly mobile) and the surface residues of proteins were flexible (that is superiorly mobile). Here, we used the temperature factor of *C*_*α*_ atom to represent the flexibility of each residue. The temperature factor was calculated according to the following equation:

1 $$ N{B}_r=\left({B}_r-\overline{B}\right)/\sigma (B) $$

Among the above equation, *B*_*r*_ represents the temperature factor of the *C*_*α*_ atom in the *γ* residue, $\overline{B}$ and *σ*(*B*) represent the mean and standard deviation of the temperature factor in the protein chain where the *γ* residue locates, respectively.

#### Thirty-six features based on the structural information of proteins

By using PSAIA [38] program, we calculated 36 structural features including solvent accessible surface area (SASA) [42], relative accessible surface area (RASA) [38], depth index (DI) of residues [43] and protrusion index (PI) of residues [44]. For SASA and RASA, we calculated 5 different values of residues: total, backbone, side-chain, polar and non-polar. For DI and PI, we calculated 4 different attribute values of residues: total mean, side-chain mean, highest and lowest. Simultaneously, we calculated the quantitative values of these structural attributes in both the unbound and bound states.

In all, we got 36 structural features, as described by Additional file 2: Table S4.

#### Five features related to the evolutionary conservation of residues

The evolutionary conservation of residues has been extensively used in studying the structures and functions of proteins. In Xia et al.’s work [24], they thought the conservation of residues were not helpful to predict hot spots. To further test the effect of residue conservation, we represented conservation in 5 different forms. By using the ConSurf server [34], we obtained two files (consurf.grades and msa_aa_variety_percentage.csv) for each protein chain in our data sets. We got the conservation score of each residue from the file consurf.grades, and calculated 4 other features based on the file msa_aa_variety_percentage.csv. In the file, it shows a table details the residues variety in percentage for each position in the query sequence. Each column shows the percentage (probability) of that amino acid found in the position in the MSA (multiple sequence alignment). So, we defined two kinds of relative conservation as follow:

2 $$ CNSV\_ REL1=\frac{{\widehat{P}}_{ra}}{{\widehat{P}}_A} $$

3 $$ CNSV\_ REL2=\frac{{\widehat{P}}_{rm}}{{\widehat{P}}_A} $$

where, ${\widehat{P}}_x={P}_x+1$, *P*_*x*_ is the percentage of residue type *x* on the certain sequence position, we do the correction by plus 1 to make sure the percentage not equals to 0. *P*_*A*_ is the percentage of the residue type “alanine” on the certain sequence position. Label ‘rm’ means the residue type with the maximum percentage, and ‘ra’ means the actual residue type on that sequence position. We also calculated the logarithm values of *CNSV*_*REL*1 and *CNSV*_*REL*2 as another two features named as *logCNSV*_*REL*1 and *logCNSV*_*REL*2, because we are not sure which representation of conservation would be more effective to differentiate hotspot residues and non-hotspot residues.

#### Thirty features related to solvent accessible area differences between the unbound and bound states

Solvent accessible surface areas (SASA) of residues have been used to predict hotspot residues in several previous studies [19, 21, 23–26]. The buried SASA is the SASA difference between the unbound and bound states. In molecular mechanics force field, the buried SASA have been considered related to desolvation energy. We suppose that different powers of buried solvent accessible surface area may correlate with different binding energy terms and thus further related to hot spots in protein-protein interfaces. We calculated 3 kinds of buried SASA and 3 kinds of buried relative SASA (RSASA) that included total SASA, polar SASA, non-polar SASA, total RSASA, polar RSASA and non-polar RSASA. In addition, we calculated different powers (1/2, 1, 3/2, 2 and 5/2) of the 6 different kinds of buried SASA, respectively. Overall, we gained 30 features for SASA.

### Feature selection

For the dataset with small size in this study, the generated 82 features can be considered high-dimensional feature space. It is necessary to conduct the feature selection to extract the effective feature subspace. In our study, we first compared 3 different feature selection methods: F-score [32], mRMR [30] and decision tree [31], the former two are filter approaches, and the latter one is a wrapper approach. Then, we proposed a hybrid feature selection strategy to select a feature subset for building the final model.

#### Decision tree

The famous decision tree algorithm was proposed by Quinlan [31]. A decision tree is a series of Boolean tests for the input pattern, and then decided the categories of the pattern. For each test, one best feature will be selected based on information gain or Gini index or others to divide the current data set. This process is repeated recursively until certain conditions are satisfied. In the present study, we used the Treefit function in MATLAB to select a subset of all features. The relevancy of different features is based on the distances between corresponding nodes and the root node.

#### F-score

F-score [32] is a simple technique that measures the discriminatory ability of two sets of real numbers. Given a data set with *n*_−_ negative examples and *n*_+_ positive examples, the F-score of feature *i* can be calculated by the following formula:

4 $$ F(i)=\frac{\left|{\overline{x}}_i^{\left(-\right)}-{\overline{x}}_i^{\left(+\right)}\right|}{\sigma_i^{\left(-\right)}+{\sigma}_i^{\left(+\right)}} $$

Where, ${\overline{x}}_i^{\left(-\right)}$ and ${\overline{x}}_i^{\left(+\right)}$ represent the averages of the *i*th feature of negative and positive examples, respectively, and ${\sigma}_i^{\left(-\right)}$ and ${\sigma}_i^{\left(+\right)}$ are the corresponding standard deviation. According to the equation, the larger the F-score is, the more powerful discrimination the feature is.

#### mRMR(maximum relevance minimum redundancy)

The mRMR is a feature selection approach first developed by Peng et al. [30], the method ranks features not only taking the relevance between features and labels into account, but also considering the redundancy among features. The relevance and redundancy in mRMR is quantified by the mutual information (MI). The MI evaluates the relationship between two vectors, which is defined by the following formula:

5 $$ I\left(x,y\right)=\iint p\left(x,y\right)\log \frac{p\left(x,y\right)}{p(x)p(y)} dxdy $$

where *x* and *y* are vectors, and *p*(*x*, *y*) is the joint probabilistic density, *p*(*x*), *p*(*y*) are the marginal probabilistic densities.

Considering the Ω_*s*_ as the subset of selected features, and Ω_*r*_ as the subset of remaining ones. If *f*_*j*_ is a feature in Ω_*r*_, *f*_*i*_ is a feature in Ω_*s*_ and *l* is the labels for all the instances, then the mRMR approach tries to select one new feature from Ω_*r*_ by the following formula:

6 $$ \underset{f_j\in {\Omega}_r}{\max}\left[I\left({f}_j,l\right)-\frac{1}{\left|{\Omega}_s\right|}{\sum}_{f_i\in {\Omega}_s}I\left({f}_j,{f}_i\right)\right] $$

In the formula, the former term is the relevance between feature *f*_*j*_ and labels *l*. The latter term is the redundancy between feature *f*_*j*_ and the features in Ω_*s*_, which is the average of the MI between feature *f*_*j*_ and the features in Ω_*s*_. The method recursively repeats this process until all features are selected, and the better feature is selected earlier.

#### Hybrid comprehensive feature selection method

**Fig. 1:**
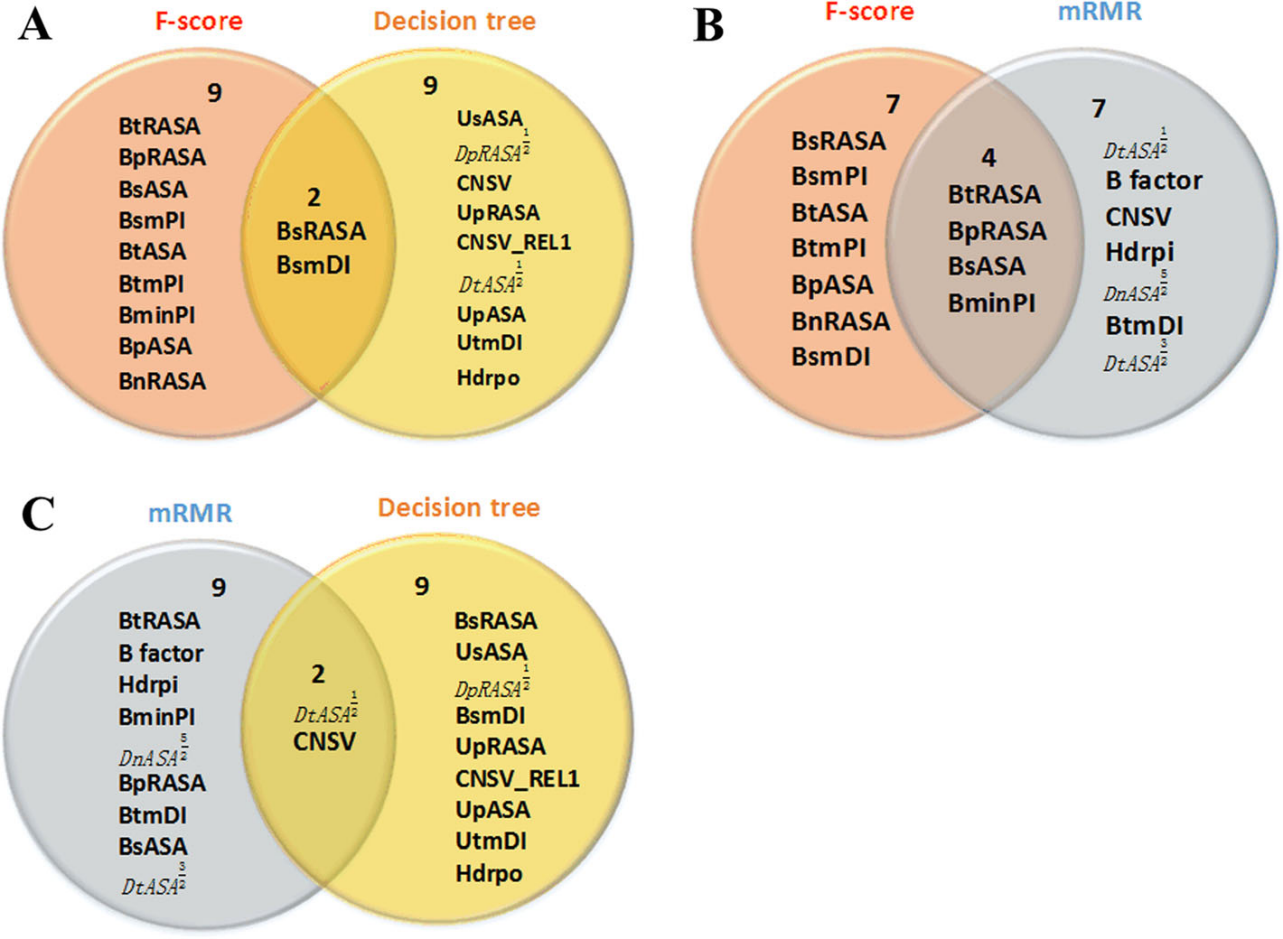
The common and different features selected by three different methods. **a** The features selected by Decision tree and F-score; (**b**) The features selected by F-score and mRMR. **c** The features selected by mRMR and Decision tree

**Fig. 2:**
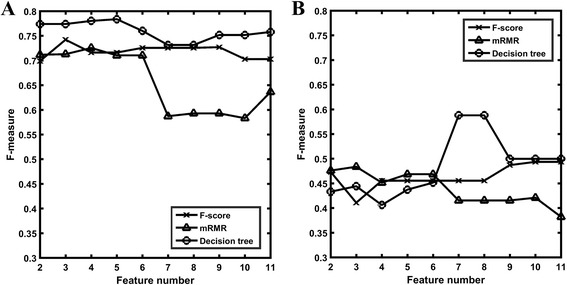
The F-measures based on different number of features selected by different methods. **a** F-measures on the cross validation tests; **b** F-measures on the independent test set

**Fig. 3:**
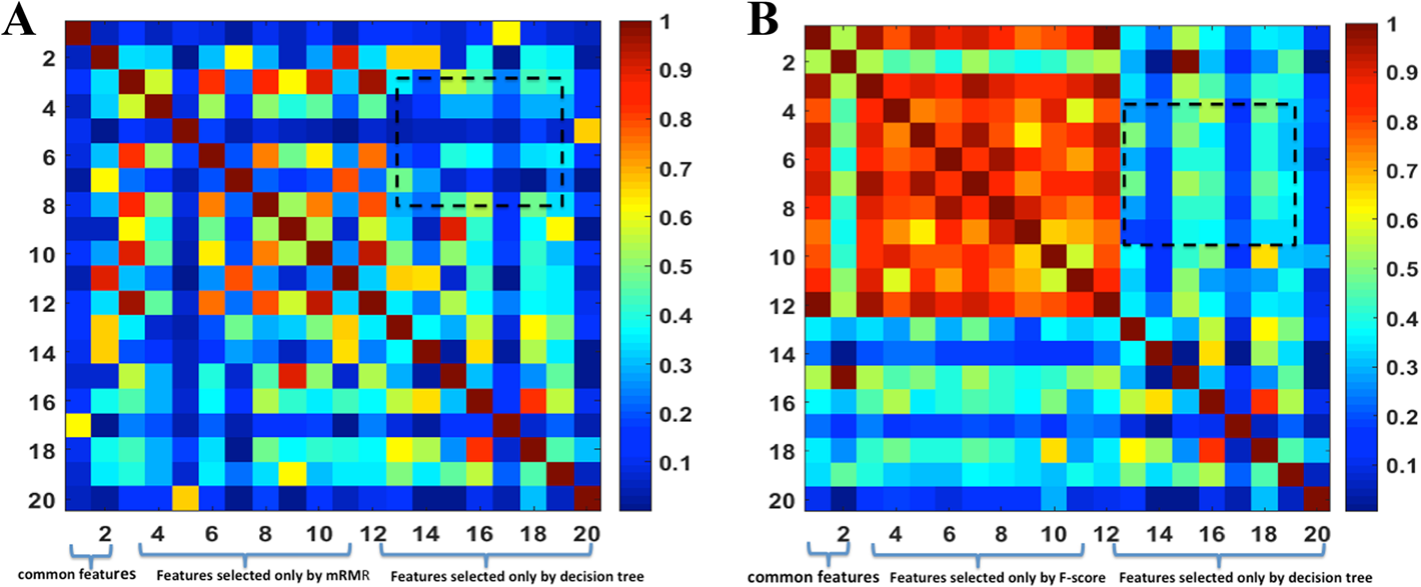
The correlation coefficient between features selected by Decision tree, mRMR, F-score for training data set. **a** Features selected by mRMR and Decision tree; **b** Features selected by F-score and Decision tree

**Fig. 4:**
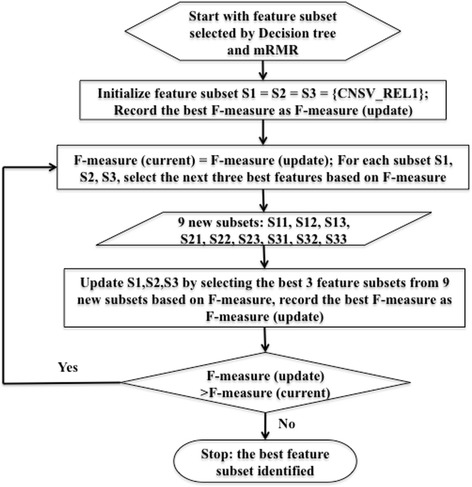
The flowchart of the hybrid feature selection strategy

We compared the features selected by Decision tree, F-score, and mRMR, indicating that there were common features and different features (Fig. 1). We also compared the predictive results based on the features selected by the three methods (Fig. 2). Although the models based on the features selected by decision tree gave better predictive performance, the features selected by decision tree and other methods could complement each other according to the principles of the methods. The correlation coefficients of the features (Fig. 3) selected by different methods also show the complementarity. We combined the features selected by the decision tree and mRMR as a feature subset. Then we used a pseudo sequential forward selection (PSFS) method to determine the optimal feature combination from the feature subset. We denoted it as pseudo SFS (PSFS) for the first feature is specified as *CNSV*_*REL*1, because the predictive accuracy on the independent test set increased substantially when the feature is added (Fig. 2b). In addition, we selected the top three feature combinations of each round for the next round. Figure 4 shows the flowchart of our hybrid feature selection process.

### Model construction

#### Support vector machine (SVM)

Support vector machine (SVM) was first proposed by Vapnik [45] and has been one of the most popular classification techniques in bioinformatics applications. It has been used for differentiating HS and NS in several previous works [21, 24–26]. In present study, we used the program LIBSVM [46] to build our models based on selected features. Different kernel functions can be used in SVM training, the radial basis function was selected in this study. For the radial basis function referring to two parameters G and C, we tried different G values (from 0 to 2) and different C values (from 0 to 40) to get the best parameter combination. In previous works [21, 23–26], different cross-validation strategies, such as leave-one-protein-out cross validation, 10 folds cross validation, and standard leave-one-out cross validation, have been used to avoid over-fitting and evaluate the predictive accuracy for the training data set. However, it has been showed that the cross validation results by different strategies were similar according to our previous work [25], so we used the standard leave-one-out cross validation in this study.

#### Model evaluation parameters

To evaluate the performance of the classification models, we calculated 5 different parameters: specificity, recall, precision, accuracy and F-measure. The 5 parameters are defined as follows:

7 $$ Specificity=\frac{TN}{TN+ FP} $$

8 $$ Recall=\frac{TP}{TP+ FN} $$

9 $$ Precision=\frac{TP}{TP+ FP} $$

10 $$ Accuracy=\frac{TP+ TN}{TP+ FP+ TN+ FN} $$

11 $$ F- measure=\frac{2 TP}{2 TP+ FP+ FN} $$

where, TP, FP, TN and FN represent the numbers of true positive (predicted hot spot residues are actual hot spots), false positive (predicted hot spot residues are actual non hot spots), true negative (predicted non hot spot residues are actual non hot spots) and false negative (predicted non hot spot residues are actual hot spots).

## Results and discussion

### Comparison of different feature selection methods

As mentioned above, we first compared three different kinds of feature selection methods: decision tree, F-score, and mRMR. Because 11 features were selected by decision tree, we selected the top 11 features ranked by F-score and mRMR as showed in Table 2. Figure 1a shows that two features (BsRASA, BsmDI) are the shared ones, which were selected by both decision tree and F-score. Figure 1b shows that four features (BtRASA, BpRASA, BsASA, BminPI) are common between the features selected by F-score and mRMR. Figure 1c shows that features (*DtASA*^1/2^, CNSV) are common between the features selected by decision tree and mRMR. It was indicated that decision tree identified more specific features that were not selected by the other two methods. Moreover, it is worth noting that the novel feature of the relative conservation (*CNSV*_*REL*1) proposed here was selected by decision tree.

**Table 2:** The top 11 features selected by three different methods

No.	Decision tree^a^	F-score	mRMR
1	BsRASA(37)	BsRASA(37)	BtRASA(35)
2	UsASA (14)	BtRASA(35)	*DtASA*^1/2^(48)
3	*DpRASA*^1/2^(52)	BpRASA (38)	B factor (11)
4	BsmDI (41)	BsASA (32)	CNSV (78)
5	CNSV (78)	BsmPI (45)	Hdrpi (5)
6	UpRASA (20)	BtASA (30)	BminPI (47)
7	*CNSV*_*REL*1 (79)	BtmPI (44)	*DnASA*^5/2^(74)
8	*DtASA*^1/2^(48)	BminPI (47)	BpRASA (38)
9	UpASA (15)	BpASA (33)	BtmDI (40)
10	UtmDI (22)	BnRASA (39)	BsASA (32)
11	Hdrpo (4)	BsmDI (41)	*DtASA*^3/2^((60)

^a^The numbers in the parentheses columns 2–4 are the feature number in the (See Additional file 2: Table S4)

Then we compared the predictive performance of the models built based on the various features selected by these three feature selection methods. Figure 2a shows the F-measures of the cross-validation for the best models built based on top 2, top 3, …, and top 11 features selected by different methods. For each feature selection methods, the increasing number of features does not guarantee the better classification performances, since they may have higher possibility of being correlated or redundancy, or result in over-fitting. Comparing the F-measures obtained by different feature selection methods, it is shown that the model performance based on the features selected by decision tree was better than that of the other two methods, with the highest F-measure of 0.784 by decision tree, in comparison with F-measures of 0.742 and 0.726 by F-score and mRMR, respectively.

In addition, we also compared the model performances of the three feature selection methods on the independent test set. Figure 2b shows the F-measures of the best models built based on top 2, top3, …, and top 11 features selected by different methods. The models based on features selected by decision tree have the highest F-measure of 0.588, which is better than the highest F-measures of the other two feature selection methods (0.494 and 0.484 for F-score and mRMR, respectively). From the curve for decision tree, it is observed that the 7th feature of the selected features substantially increased the classification performance, which is *CNSV*_*REL*1 that is a unique feature introduced in this work.

### Model based on hybrid comprehensive feature selection

By comparing the features selected by the above three different methods, we inferred that there exists complementarity among the selected features of different selection methods. Figure 3a shows the correlation coefficients between the features selected by mRMR and decision tree. It shows that the correlation coefficients between the features selected only by mRMR and correlation coefficients between the features selected only by decision tree are generally higher than the correlation coefficients between the features selected by the two different methods (dashed square in Fig. 3a). Figure 3b shows the correlation coefficients between the features selected by F-score and decision tree. It shows that the correlation coefficients between the features selected only by F-score and correlation coefficients between the features selected only by decision tree are generally higher than the correlation coefficients between the features selected by the two different methods (dashed square in Fig. 3b). It is also indicated that the several features selected by F-score were highly correlated. In addition, the correlation coefficients between features selected by mRMR and decision tree were generally lower than the features selected by F-score and decision tree.

**Fig. 5:**
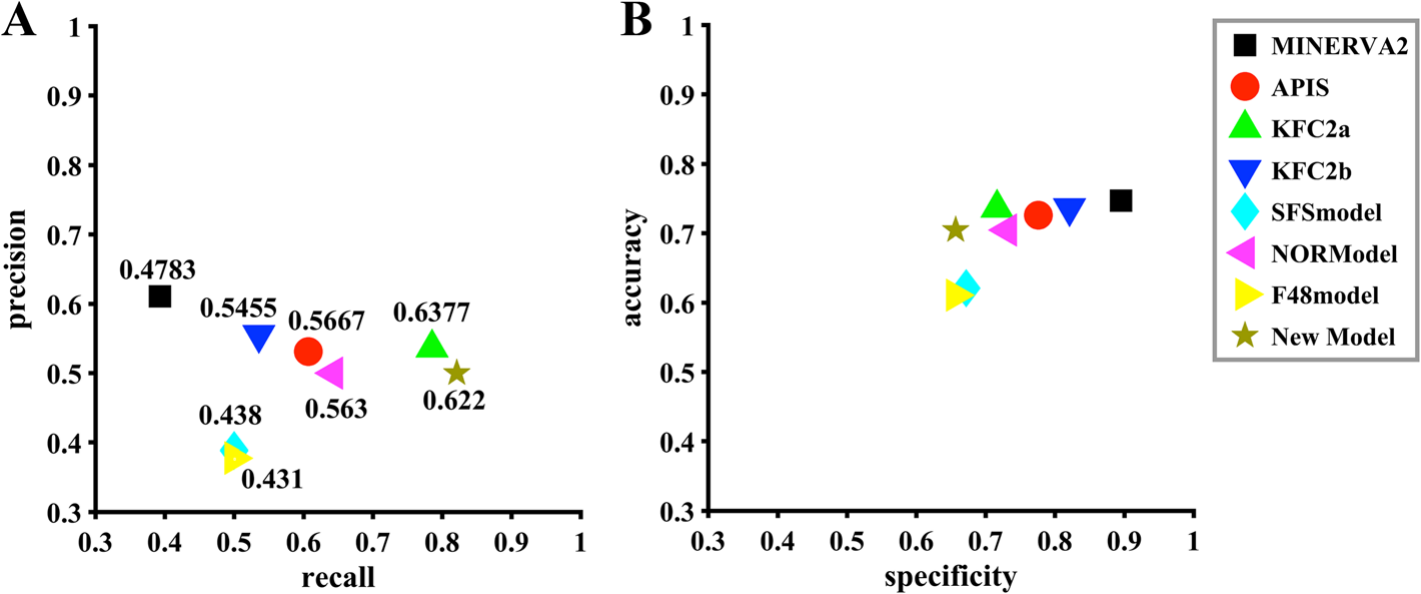
Comparison of models used for prediction of the hot spots in terms of the five parameters for the independent test set. **a** Predictive evaluation in terms of Precision-Recall and F-measure; **b** Predictive evaluation in terms of Accuracy-Specificity

**Fig. 6:**
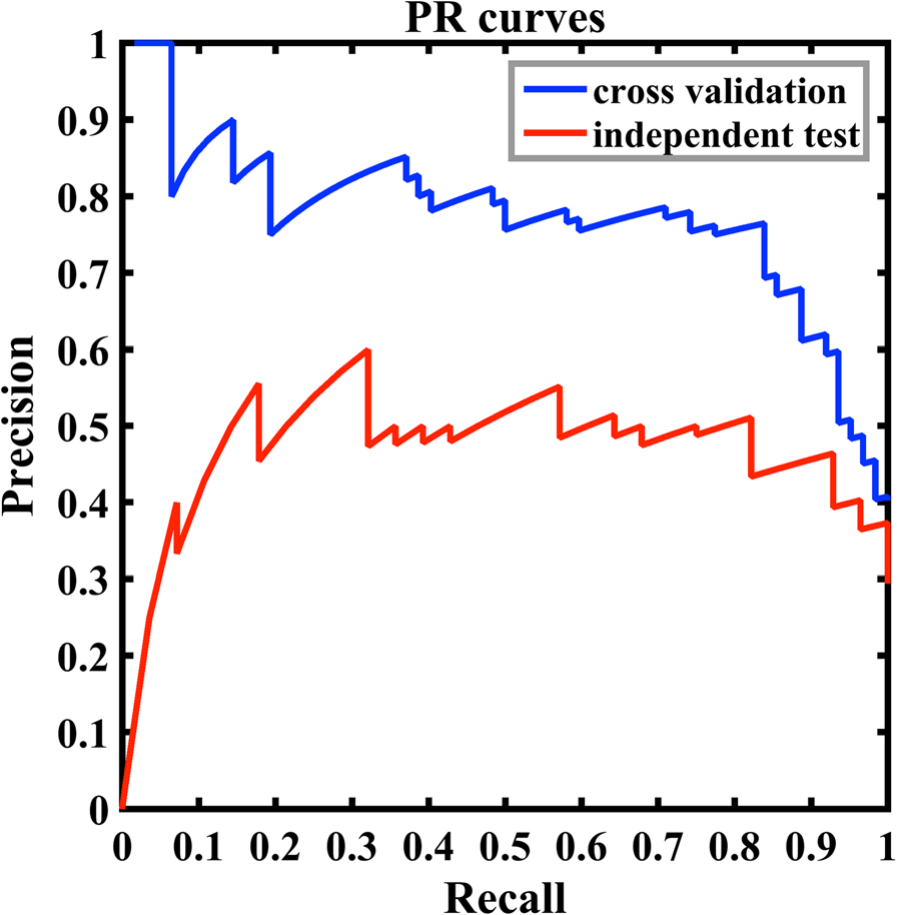
The P-R curves for cross-validation results of the training data set and the predictive results of the independent test set

Therefore, we combined the features selected by decision tree and mRMR as a feature subset. The feature subset is shown in Table 3. Then we used a PSFS method to identify the best feature combination by using *CNSV*_*REL*1 as the initial feature. Table 4 shows the selected features by our classifier (one feature was added at a time or round) and the corresponding cross-validation performance in each round. The results show that the predictive performances are convergent at the 5th round. The best F-measure is 0.800. In round 5, two feature combinations show the best predictive performances, which are feature combination including BsRASA, CNSV_REL1, *DtASA*^1/2^, UpASA, BtRASA, and B factor and feature combination including BsRASA, CNSV_REL1, *DtASA*^1/2^, UpASA, BtRASA, and BtmDI. The feature BtmDI is the bound total mean depth index. B factor can be calculated directly from PDB file, but the bound total mean depth index was calculated by program PSAIA. Therefore, we constructed our final model based on the former feature combination. Based on these 6 features, our model achieved 0.800, 0.831, 0.839, and 0.765 for F-measure, accuracy, recall and precision respectively for cross validation on training data set (Table 4). The model was built with parameters G = 0.001 and C = 4.0. On the independent test set, our model achieved 0.622, 0.705, 0.821, and 0.500 for F-measure, accuracy, recall, and precision, respectively (Fig. 5). Since F-measure is a harmonic average of precision and recall, we also plot the P-R curve for our model. As showed in Fig. 6, we plot the curves both for cross validation results on the training data set and the test results on the independent test set. It shows distinct differences between the two curves. The reason is “whether the example with the largest output value is positive or negative greatly changes the PR curve (approaching (0,1) if positive and (0,0) if negative)” [47]. In addition, the empirical P-R curves are highly imprecise estimate of the true curve, especially in the case of a small sample size and the class imbalance in favor of negative examples [48]. So we also plot the ROC curves here as an alternative of P-R curves. As showed in Additional file 2: figure S1, the areas under the ROC curves are 0.853 and 0.764 for cross-validation and independent test, respectively.

**Table 3:** The feature subset combined the features selected by decision tree and mRMR

Feature abbreviation	Feature full name
BsRASA	Bound side-chain relative accessible surface area
UsASA	Unbound side-chain accessible surface area
*DpRASA* ^1/2^	${\left({RASA}_{unb}(polar)-{RASA}_{bnd}(polar)\right)}^{\frac{1}{2}}$
BsmDI	Bound side-chain mean depth index
Conservation	Conservation
UpRASA	Unbound polar relative accessible surface area
CNSV_REL1	$CNSV\_ REL1=\frac{{\widehat{P}}_{ra}}{{\widehat{P}}_A}$
*DtASA* ^1/2^	${\left({ASA}_{unb}(total)-{ASA}_{bnd}(total)\right)}^{\frac{1}{2}}$
UpASA	Unbound polar accessible surface area
UtmDI	Unbound total mean depth index
Hdrpo	Hydrophobicity
BtRASA	Bound total relative accessible surface area
B factor	Temperature factor
Hdrpi	Hydrophilicity
BminPI	Bound minimal protrusion index
*DnASA* ^5/2^	${\left({ASA}_{unb}\left( non- polar\right)-{ASA}_{bnd}\left( non- polar\right)\right)}^{\frac{5}{2}}$
BpRASA	Bound polar relative accessible surface area
BtmDI	Bound total mean depth index
BsASA	Bound side-chain accessible surface area
*DtASA* ^3/2^	${\left({ASA}_{unb}(total)-{ASA}_{bnd}(total)\right)}^{\frac{3}{2}}$

**Table 4:** Features selected and the corresponding cross-validation performance in PSFS process

Round	Features identified	Accuracy	Recall	Precision	F-measure
1	CNSV_REL1, BsRASA	0.766	0.790	0.681	0.731
	CNSV_REL1, BpRASA	0.779	0.710	0.733	0.721
	CNSV_REL1, BtRASA	0.753	0.694	0.694	0.694
2	CNSV_REL1, BsRASA, UsASA	0.818	0.774	0.774	0.774
	CNSV_REL1, BtRASA, UpASA	0.799	0.790	0.731	0.760
	CNSV_REL1, BsRASA, UpASA	0.799	0.774	0.739	0.756
3	CNSV_REL1, BsRASA, UpASA, BtRASA	0.818	0.807	0.758	0.781
	CNSV_REL1, BtRASA, UpASA, *DtASA*^1/2^	0.812	0.807	0.746	0.775
	CNSV_REL1, BsRASA, UpASA, BpRASA	0.812	0.807	0.746	0.775
4	CNSV_REL1, BtRASA, UpASA, *DtASA*^1/2^, BsRASA	0.825	0.838	0.754	0.794
	CNSV_REL1, BsRASA, UpASA, BtRASA, *DpRASA*^1/2^	0.818	0.823	0.750	0.785
	CNSV_REL1, BsRASA, UpASA, BpRASA, *DtASA*^1/2^	0.825	0.823	0.761	0.791
5	CNSV_REL1, BtRASA, UpASA, *DtASA*^1/2^, BsRASA, BtmDI	0.831	0.839	0.765	0.800
	CNSV_REL1, BtRASA, UpASA, *DtASA*^1/2^, BsRASA, B factor	0.831	0.839	0.765	0.800
	CNSV_REL1, BsRASA, UpASA, BpRASA, *DtASA*^1/2^, BtmDI	0.825	0.823	0.761	0.791
6	CNSV_REL1, BtRASA, UpASA, *DtASA*^1/2^, BsRASA, B factor, BtmDI	0.831	0.839	0.765	0.800
	CNSV_REL1, BtRASA, UpASA, *DtASA*^1/2^, BsRASA, BtmDI, Hdrpi	0.831	0.839	0.765	0.800
	CNSV_REL1, BtRASA, UpASA, *DtASA*^1/2^, BsRASA, B factor, BminPI	0.831	0.839	0.765	0.800

To further prove the effect of our hybrid feature selection method, we compared the performance of our model with the model built based on features selected only by sequential forward selection (SFS) method. We performed a SFS on the entire set of 82 features. Additional file 2: Table S6 shows the features selected in different rounds. The final model, named SFSmodel, was built based on feature combination including BbRASA, BpRASA, BnRASA, DpASA, *DnASA*^3/2^. Although Table S6 shows the F-measure of SFSmodel for cross validation is 0.832 that is higher than the F-measure of our model for cross validation (0.800), the F-measure of SFSmodel on the independent test set is 0.438 (showed in Fig. 5) that is substantially lower than the F-measure of our model (0.622). This proved that our hybrid feature selection method is more effective than SFS in our study.

According to the results stated in this section and the former section, we shown that the model based on the features selected by our hybrid feature selection strategy outperformed the models based on the other four feature selection methods.

### Comparison with other methods on the independent data set

To further evaluate the performance of our model, we compared the predictive results of our model with several other hot spot prediction methods such as MINERVA2 [21], APIS [24], KFC2 [25]. In MINERVA2, the authors presented 54 features including atom contacts, density, hydrophobicity, surface area burial, residue conservation and so on. They used decision tree to select the relevant feature subset and built their models using SVM. In APIS, the authors presented 62 features including physicochemical features of residues, protein structure features generated by PSAIA, pairwise residue potential, residue evolutionary rate and temperature factor. They used the F-score to select the relevant features and built their models also using SVM. Their final model APIS is a combined model based on protrusion index and solvent accessibility. In KFC2, the authors presented 47 different features including solvent accessibility, atomic density, plasticity features and so on. They considered the feature combinations of different feature numbers and did a thoroughly search to obtain the final feature subset. Then they built their models by using SVM. In our study, we presented 82 different features including some new proposed features such as the relative residue conservation (CNSV_REL1, CNSV_REL2, *logCNSV*_*REL*1 and *logCNSV*_*REL*2) and the different powers of the buried solvent accessible surface area. As the feature selection is important for building the models, we proposed a hybrid feature selection process to select the effective feature subset by combined mRMR, decision tree and PSFS. As showed in Fig. 5, our method has the highest recall (0.821) among all the methods, and the F-measure (0.622) of our method is comparable to KFC2a that shows the highest value (0.638) among all the methods. A high recall means the method can identify most of the interface hotspots, which is meaningful for experimental scientists. In addition, F-measure is a robust evaluation measure for both positive and negative instances especially for the imbalanced data sets such as our training data set and the independent test set. The F-measure of our method is higher than other methods and comparable to the method KFC2a with the highest value.

Although our model does not show superior values for the rest three parameters (accuracy, specificity, and precision), the three parameters are not independent. In this study, the dataset is imbalanced for having more negative examples, so high specificity often means high accuracy. Specificity is used to evaluate the predictive accuracy of negative examples, however, precision is related to specificity. According to the less positive examples in our dataset, high precision often means low false positives, which means the high specificity. On the other hand, recall is only used to evaluate the predictive accuracy of positive examples. So, we considered recall and precision as two basic criteria for evaluating the performances of different models. High recall means that a model returned most of the relevant examples, while high precision means that a model returned more relevant examples than irrelevant ones. Since it is hard for a classification method to have both high precision and recall, we can divide those methods into two types: high recall methods and high precision methods. Our model shows the highest recall among all the methods compared in Fig. 5. It provides a choice for users to select the model based on their goals. For the overall performance, it is better to use an integrated parameter such as F-measure to do the evaluation.

Besides, in biological study, researchers often want to understand the mechanism of protein-protein binding. Hotspot residues can provide corresponding clues, and the more hotspots are identified, the more accurately the mechanism will be understood. Our model shows the highest recall which means the highest coverage of the hotspot residues.

In MINERVA2 and APIS, both of the authors thought the residue conservation is not good for identifying hot spots, however, our results indicated that the relative residue conservation could be an effective feature for predicting hot spot residues. In addition, the authors of MINERVA2 used decision tree to select the effective subset. Decision tree carries out a greedy search process to choose feature to discriminate examples, it possibly introduces more features, some of which are irrelevant. The authors of APIS used F-score to select the relevant feature subset. The correlation between features and labels are considered in F-score, however, the correlation between the features is not considered. We proposed the hybrid feature selection process by combining both filter and wrapper technique. By using mRMR, and decision tree, we selected a feature subset that contains both relevant and complementary features according to the algorithms of the two methods. Simultaneously, the size of the subset is only one fourth of the original features. Then we used a PSFS process to identify the optimal feature subset. The general sequential forward selection (SFS) algorithm easily gets in a local optimum, however, our PSFS process assigns three choices for the next round. So it has more chance to reach the global optimum.

Noticed that both Cho et al. [21] and Xia et al. [24] have tried to build their models based on the normalized features, we also built a model, named NORModel, based on normalized features by using our hybrid feature selection method. We normalized our 82 features by the Z-transform. Additional file 2: Table S7 shows the normalized features selected by decision tree, mRMR and F-score, which are the same as features selected in Table 2. Additional file 2: figure S2A shows the F-measures of the cross-validation for the best models built based on top 2, top 3, …, and top 11 normalized features selected by the three feature selection methods. Additional file 2: figure S2B shows the performances on the independent test set based on the best models built based on top 2, top3, …, and top 11 normalized features. Similarly, we observed our new feature CNSV_REL1 (the 7th feature of decision tree) could improve the performance on independent test set although not as obviously as in Fig. 2. Then we used our PSFS method to select the optimal feature combination. Table S8 shows the features selected in different rounds. The final model, named NORModel, was built based on 7 features including CNSV_REL1, UpASA, UtmDI, BtRASA, B factor, BpRASA, BtmDI. Although Additional file 2: Table S8 shows the F-measure of NORModel for cross validation is 0.825 that is higher than the F-measure of our model for cross validation (0.800), the F-measure of NORModel for independent test set is 0.563 (showed in Fig. 5), that is substantially lower than the F-measure of our model (0.622). Our study indicates that the model built on normalized features does not necessarily have better performances than the model built on non-normalized features.

In addition, we analyzed if our model can complement other methods. Overall, our model has the most overlap (80% common prediction) with KFC2a and the least overlap (65.3% common prediction) with MINERVA2. By combining our model with MINERVA2, we got a F-measure of 0.6316 on the independent test set, which is a little bit higher than our model (0.622). The way we combined two models is if any one of the two models predicts a residue as hotspot, the combined model predicts the residue as hotspot, otherwise the residue is predicted as non-hotspot. The predictive performance by combining any two methods are showed in Table S9. It turned out five of the ten combine models showed slight improvement compared with the single models according to F-measure.

### Predictive performance on the medium test set

For the medium test set that contain 98 residues with ∆∆*G* between 0.4 kcal/mol and 2.0 kcal/mol, our model predicted 32 of them as hot spot residues, 66 as non hot spot residues. Specifically, our model predicted 14 of 40 residues with ∆∆*G*≥1.2 kcal/mol as hot spot residues. By comparing with other methods, only KFC2a predicted 17 of 40 residues with ∆∆*G*≥1.2 kcal/mol as hot spot residues, which is higher than our model. Note that some of the residues (See Additional file 1: Table S2) had been used to train the KFC2a and KFC2b models.

### Predictive performance for different types of residues and different types of interfaces

To evaluate if our model is biased to predict certain types of residues or certain types of interfaces better than other types, we further analyzed the performance of our model for different types of residues and different types of interfaces. According to the physicochemical characteristics of the residues, the twenty types of residues can be categorized into charged residues, polar residues, and aromatic residues. Table 5 shows the predictive performances of our model on different types of residues, it turned out our model showed slight better performance on aromatic and non-polar residues than other types of residues according to F-measure. By the two-sample t-test, we calculated the *p*-values between charged and non-charged residues, polar and non-polar residues, and aromatic and non-aromatic residues, which are 0.400, 0.119, and 0.0158, respectively. This result indicates the performance difference of our model between aromatic and non-aromatic residues is statistically significant. The interface information was collected from SCOPPI database [49]. SCOPPI is a structural classification database of protein-protein interfaces, and their interfaces derived from PDB [50] structure files. The interfaces information referred to the interfaces in our independent test set was shown in Additional file 2: Table S10. Three interfaces, from 1CDL, 1DVA, and 1JPP, were not recorded in SCOPPI database. We first checked the performances of our model on interfaces recorded in SCOPPI and the interfaces not recorded in SCOPPI. As showed in Additional file 2: Table S11, our model showed better performance for residues in the interfaces recorded in SCOPPI than those not in SCOPPI database according to F-measure. However, the difference is not statistically significant for the p-value is 0.269. After we checked the three interfaces not recorded in SCOPPI, we found these three interfaces are between proteins and peptides. Another information we used to divide the interfaces is the interface size. The interface size is calculated as the difference of solvent accessible surface area (*∆SASA*) between the proteins in bound and unbound states. According to the definition of SCOPPI database, if ∆*SASA*≥2000Å^2^, the interface is categorized into large size, if 1400Å^2^≤∆*SASA*<2000Å^2^, the interface is categorized into medium size, otherwise the interface is categorized into small size. As showed in Additional file 2: Table S11, it turned out our model showed better performance for medium and large interfaces than small interfaces. However, the p-values between residues in small and medium size interfaces, in small and large size interfaces, and in medium and large size interfaces are 0.161, 0.213, and 0.735, respectively, which indicates the differences are not statistically significant.

**Table 5:** Statistical performance of our model for predicting hotspot of the independent test set by types of amino acids

Types of residues	Accuracy	Specificity	Recall	Precision	F-measure
All residues (28 HS/ 67 NS)	0.705	0.657	0.821	0.500	0.621
Charged residues^a^ (13 HS/ 28 NS)	0.732	0.750	0.692	0.563	0.621
Non Charged residues	0.685	0.590	0.933	0.467	0.622
Polar residues^b^ (14 HS/41 NS)	0.709	0.707	0.714	0.455	0.556
Non polar residues	0.700	0.577	0.929	0.542	0.685
Aromatic residues^c^ (11 HS/12 NS)	0.652	0.417	0.818	0.563	0.667
Non aromatic residues	0.736	0.709	0.824	0.467	0.596

^a^Charged residues: D,E,K,R,H

^b^polar residues: D,E,K,R,H,S,T,N,Q

^c^aromatic residues: F, H,W,Y

### Post analysis of the selected features of the final model

**Fig. 7:**
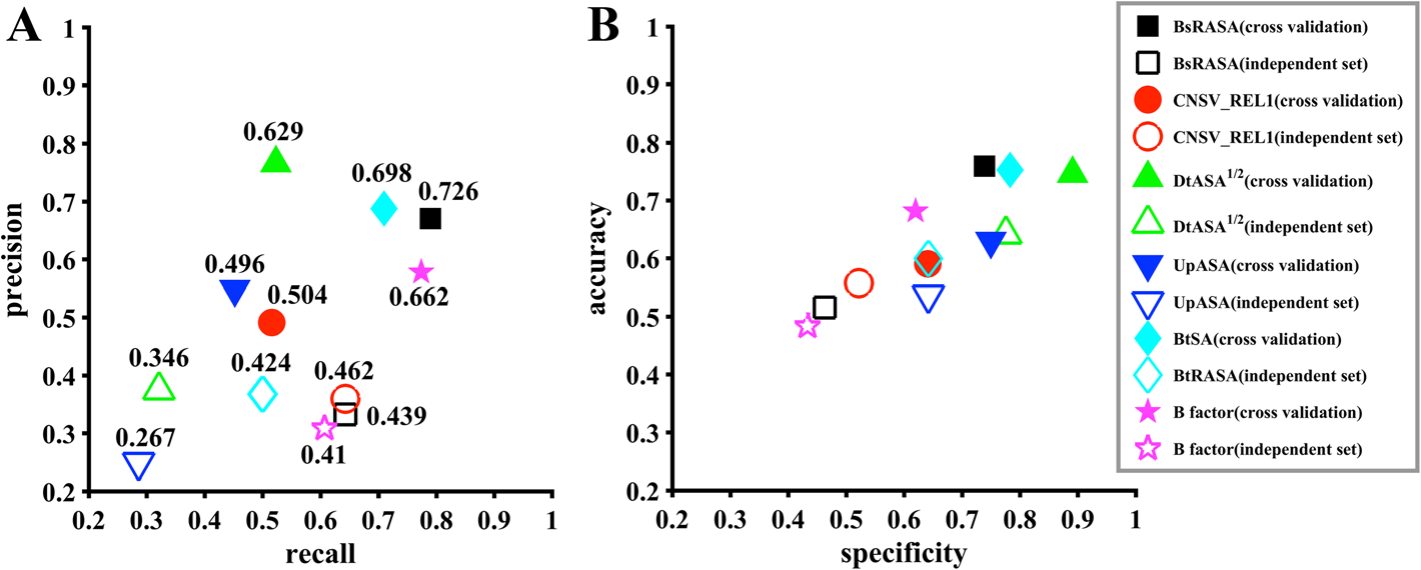
Comparison of models built based on different single features in terms of the five parameters for the independent test set. **a** Predictive evaluation in terms of Precision-Recall and F-measure; **b** Predictive evaluation in terms of Accuracy-Specificity

To evaluate the importance of the features selected by the final model, we did a post analysis of the selected features. Firstly, we investigated the predictive power of individual features. As showed in Fig. 7a, the feature CNSV_REL1 shows the best generalization of all the selected features according to the F-measure, for which the F-measures on cross validation and the independent test set are 0.504 and 0.462, respectively. However, the F-measure differences are between 0.229 and 0.287, which means models based on other features have a worse generalization compared to feature CNSV_REL1. In the feature selection section (Fig. 2b), we noticed the F-measure increased substantially when feature CNSV_REL1 was added. This means the high generalization of single feature is of benefit to the final model with combined features. This implies that we should make sure at least one feature with high generalization be selected when we do the feature selection.

In addition, we did a post analysis by removing one of the selected features and checking the performance of the models built based on the remaining features. As showed in Table 6, when we removed the feature BsRASA, CNSV_REL1, *DtASA*^1/2^, UpASA, BtRASA, respectively, the predictive accuracies decreased as expected. Especially, the predictive accuracies decreased substantially when CNSV_REL1 and UpASA were removed. According to the analysis above, it was not surprising that the classification performance dropped down when the CNSV_REL1 was removed. It was surprising that the performance dramatically dropped down when UpASA was removed. As showed in Fig. 7, UpASA by itself shows the worst predictive performance among all the selected features. So, we supposed the feature could supplement the other features when the model was built. As showed in Fig. 1, this feature was not selected by F-score because this method only considers the relationship between the feature and the labels. The feature was also not selected by mRMR, because this method considers the correlations between the features and the labels and the redundancy between features, but not mainly the complementarity. On the contrary, the decision tree can partially reflect the complementarity of the features to some extent.

**Table 6:** Predictive results of the models built by removing one of the selected features

Feature removed	Accuracy^a^	Specificity	Recall	Precision	F-measure
BsRASA	0.818/0.684	0.815/0.657	0.807/0.750	0.746/0.477	0.775/0.583
CNSV_REL1	0.786/0.632	0.783/0.582	0.790/0.750	0.710/0.429	0.748/0.545
*DtASA* ^1/2^	0.818/0.684	0.826/0.642	0.807/0.786	0.758/0.478	0.781/0.595
UpASA	0.786/0.632	0.815/0.627	0.742/0.643	0.730/0.419	0.736/0.507
BtRASA	0.805/0.705	0.837/0.672	0.758/0.786	0.758/0.500	0.758/0.611
B factor	0.825/0.705	0.815/0.658	0.839/0.821	0.754/0.500	0.794/0.622

^a^For columns 2–6, the values above the slashes are for cross validation, and the values under the slashes are for the independent test set

In addition, we did a two-sample T-test to test if there are significant differences of the six selected features between hotspot residues and non-hot spot residues in the training data set. We obtained *P*-values of 5.44 × 10^−12^, 3.23 × 10^−2^, 4.83 × 10^−9^, 0.612, 4.36 × 10^−11^, 6.14 × 10^−7^ for features BsRASA, CNSV_REL1, *DtASA*^1/2^, UpASA, BtRASA, B-factor, respectively. The result also shows UpASA is not a good feature by itself, but it can complement other features for differentiating hotspots from non-hotspots.

Besides, to further test the effect of our newly proposed features, we have built a model based only on the old 48 features reported in Xia et al.’s paper [24] by using our hybrid feature selection method. The model was named F48model. We first used three feature selection methods, F-score, mRMR, and decision tree, to select features from the 48 features. The results are showed in Additional file 2: Table S12. Then, we built models by using top 2, top 3, …, top10 selected features, and tested on the independent test set. The results are showed in Additional file 2: figure S3. However, no feature that can substantially improve the ability of the generalization was observed according to Additional file 2: figure S3B. So we performed our PSFS process without an initial feature, the features selected in each round of PSFS were listed in Additional file 2: Table S13. Finally, the F48model was built based on 6 features including BsRASA, UsASA, UnASA, B factor, *DtASA*^1/2^, UtmDI. The F-measure for the cross-validation is 0.836 that is higher than our model (0.800), however, the F-measure for the independent test set of F48model is 0.431 that is substantially less than our model (0.622) as showed in Fig. 5. This results proved the effect of our new proposed features.

### Case studies

**Fig. 8:**
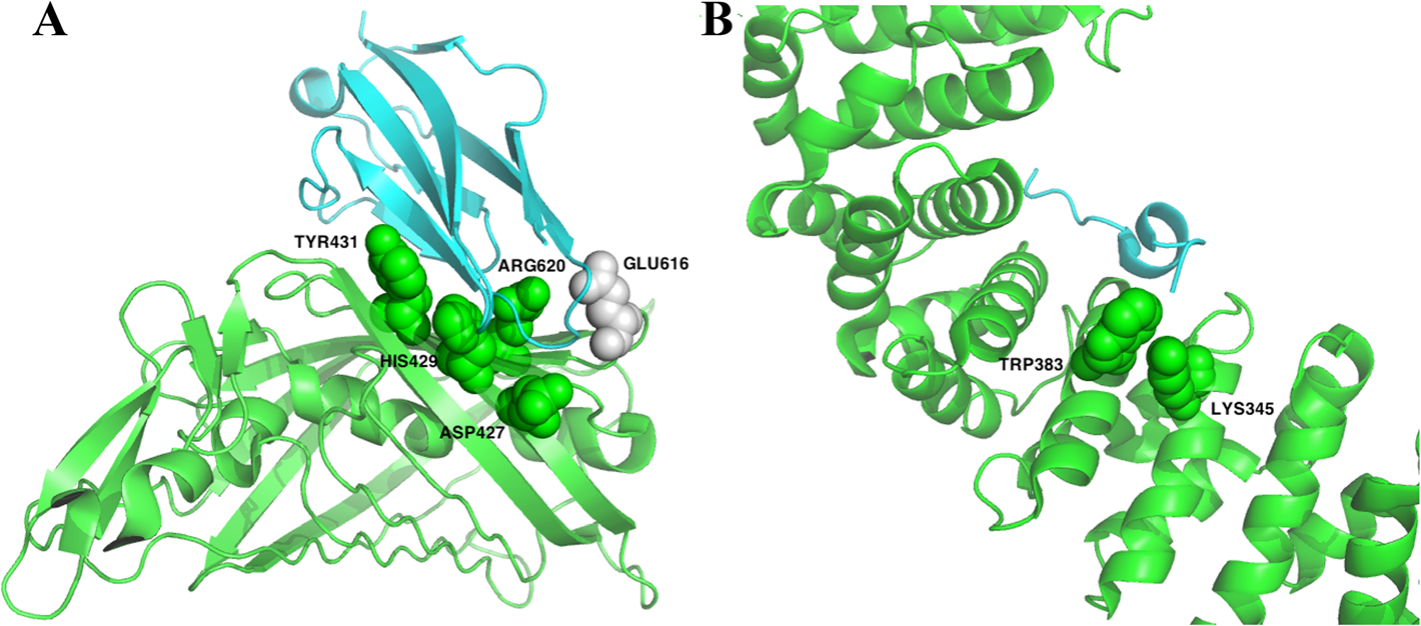
Interface hot spot residues of nidogen-1 G2 and perlecan IG3, and β-catenin and adenomatous polyposis coli protein. **a** The complex of nidogen-1 G2 and perlecan IG3 (PDB ID: 1GL4), for which 4 out of the 5 hot spot residues were identified by our model; **b** The complex of β-catenin and adenomatous polyposis coli protein (PDB ID: 1JPP), for which all of the 2 hot spot residues were identified by our model. Green sphere: true positive hot spot residues; Gray sphere: false negative hot spot residues

To visualized show the hotspot residues on the protein-protein interfaces, we plotted two cases by using PyMol. The first one is the complex of nidogen-1 G2 and perlecan IG3, for which the PDB ID is 1GL4. As showed in Fig. 8a, 5 hotspot residues at the interface had been recorded in the independent test set. Our model identified 4 of the 5 hot spot residues as hotspots, in comparison with other methods, MINERVA2, APIS, KFC2A and KFC2B predicted 2, 3, 3, 3 of the 5 hot spot residues as hotspots, respectively. The second case is the complex of β-catenin and adenomatous polyposis coli protein, for which the PDB ID is 1JPP. As showed in Fig. 8b, 2 hotspot residues at the interface had been recorded in the independent test set. Our model identified all of them, while other methods missed all of them.

## Conclusion

Hot spot residues prediction at the protein-protein interface can be helpful for experimental scientists to identify actual hot spot residues. In the last decade, a few knowledge-based computational methods had been proposed. Many different kinds of features have been used to build the models in these methods, and feature selection was an existing bothering problem when building those models. In this work, we compared three different feature selection methods including F-score, mRMR, and Decision tree. Generally, the features selected by Decision tree shows better predictability compared with those selected by the other two methods. We analyzed the correlation between features selected by mRMR and Decision tree respectively, and our results showed that the correlation coefficients between different features selected by the two methods were relatively small, which indicated that possible complementarity existed between the features selected by the two methods. According to these results, we proposed a hybrid strategy of feature selection. Firstly, we combined the features selected by both mRMR and decision tree, and then used a pseudo sequential forward selection (PSFS) process to select the best feature combination. By this strategy, first, we reduced the feature dimension. Second, we believed our feature selection process could integrate the virtues of both filter and wrapper feature selection approaches to select the features not only relevant by itself but also complementary to each other. Thus, the model built based on the feature subset selected by our strategy might show good ability for generalization. Based on the features selected by the hybrid feature selection strategy, we build a hot spots prediction model that showed a F-measure 0.622 and a recall 0.821 for the independent test set, which is better or comparable to the state-of-art hot spot prediction methods.

In addition, we did a post-analysis for the final feature combination. Firstly, we investigated the predictive performances for each of the selected features. Our newly added feature, *CNSV*_*REL*1, was found to have the best ability for generalization (F-measure 0.504 and 0.462 for cross validation and the independent test set). Then, we removed one of the selected features and checked the models built with the remaining features. After removing two features, *CNSV*_*REL*1 and UpASA, the predictive accuracy of the model was substantially decreased. We noticed that the feature UpASA had the lowest ability for generalization by itself, which indicates that the complementarity between features is important for the final model. To conclude, both generalization of the single feature and the complementarity between features are important and should be considered in feature selection methods.

## Additional files

Additional file 1: Datasets for Protein-protein interface hot spots prediction based on a hybrid feature selection strategy. This file provides more detailed data for the data sets. **Table S1.** The interface residues with observed *∆∆G* values of the training data set. **Table S2.** The interface residues with observed *∆∆G* values of the medium test set. **Table S3.** Data set of interface residues and hot spot predictions for the independent test set. (XLS 72 kb)

Additional file 2: Supplementary Information for Protein-protein interface hot spots prediction based on a hybrid feature selection strategy. This file provides all the features generated in this study, and other tables for analysis and discussion. **Table S4.** All 82 features generated in the study. **Table S5.** The numerical values of 10 different kinds of properties of the 20 amino acids. **Table S6.** Features selected from 82 features and the corresponding cross validation performance in SFS process. **Table S7.** The top 11 normalized features selected by decision tree, F-score and mRMR. **Table S8.** Features selected and the corresponding cross-validation performance in PSFS process for normalized features. **Table S9.** Consensus results based on combining any two of the five models (MINERVA2, APIS, KFC2a, KFC2b, Our model). **Table S10.** Interface information referred to the interfaces in the independent test set. **Table S11.** Statistical performance of our model for predicting hotspot of the independent test set by the types of protein-protein interfaces. **Table S12.** The top 10 features selected by decision tree, F-score and mRMR. **Table S13.** Features selected and the corresponding cross-validation performance in PSFS process for the 48 old features reported in Xia et al.’s paper. **Figure S1.** The ROC curves for cross-validation results of the training data set and the predictive results of the independent test set. **Figure S2.** The F-measures based on different number of normalized features selected by different methods. A. F-measures on the cross validation; B. F-measures on the independent test set. **Figure S3.** The F-measures based on different number of features of the 48 old features selected by different methods. A. F-measures on the cross validation; B. F-measures on the independent test set. (PDF 10501 kb)

## Abbreviations

HS: Hot Spot
mRMR: maximum Relevance Minimum Redundancy
NS: Non-hot Spot
PSFS: Pseudo Sequential Forward Selection
SASA: Solvent Accessible Surface Area
SFS: Sequential Forward Selection
SVM: Support Vector Machine
