# Supplementary material for: Protein-protein interface hot spots prediction based on a hybrid feature selection strategy
Source: BMC Bioinformatics. 2018 Jan 15;19:14. doi: 10.1186/s12859-018-2009-5 (PMC5769548; doi:10.1186/s12859-018-2009-5)
Supplement: Supplementary file 2 — Supplementary Information for Protein-protein interface hot spots prediction based on a hybrid feature selection strategy. This file provides all the features generated in this study, and other tables for analysis and discussion. Table S4. All 82 features generated in the study. Table S5. The numerical values of 10 different kinds of properties of the 20 amino acids. Table S6. Features selected from 82 features and the corresponding cross validation performance in SFS process. Table S7. The top 11 normalized features selected by decision tree, F-score and mRMR. Table S8. Features selected and the corresponding cross-validation performance in PSFS process for normalized features. Table S9. Consensus results based on combining any two of the five models (MINERVA2, APIS, KFC2a, KFC2b, Our model). Table S10. Interface information referred to the interfaces in the independent test set. Table S11. Statistical performance of our model for predicting hotspot of the independent test set by the types of protein-protein interfaces. Table S12. The top 10 features selected by decision tree, F-score and mRMR. Table S13. Features selected and the corresponding cross-validation performance in PSFS process for the 48 old features reported in Xia et al.’s paper. Figure S1. The ROC curves for cross-validation results of the training data set and the predictive results of the independent test set. Figure S2. The F-measures based on different number of normalized features selected by different methods. A. F-measures on the cross validation; B. F-measures on the independent test set. Figure S3. The F-measures based on different number of features of the 48 old features selected by different methods. A. F-measures on the cross validation; B. F-measures on the independent test set. (PDF 10501 kb) [file 12859_2018_2009_MOESM2_ESM.pdf]

Supplementary Information for

Protein-protein interface hot spots prediction based on a hybrid feature selection strategy

Yanhua Qiao[1], Yi Xiong[2, 3], Hongyun Gao[4], Xiaolei Zhu[1*], and Peng Chen[5*]

[1] School of Life Sciences, Anhui University, Hefei, Anhui 230601, China

[2] State Key Laboratory of Microbial Metabolism, Shanghai JiaoTong University, Shanghai 200240, China

[3] School of Life Sciences and Biotechnology, Shanghai JiaoTong University, Shanghai 200240, China

[4] Information and Engineering College, Dalian University, Dalian, Liaoning 116622, China

[5] Institute of Health Sciences, Anhui University, Hefei, Anhui 230601, China

Contact: xlzhu_mdl@hotmail.com; pchen@ahu.edu.cn

Table S4. All 82 features generated in the study.

No.	Feature description	Symbol
1	Number of atoms	Na
2	Number of electrostatic charge	Nec
3	Number of potential hydrogen bonds	Nphb
4	Hydrophobicity	Hdrpo
5	Hydrophilicity	Hdrpi
6	Propensity	Prop
7	Isoelectric point	Isoep
8	Mass	Mass
9	Expected number of contacts within 14Å sphere	Enc
10	Electron-ion interaction potential	Eiip
11	Temperature factor	B factor
12	Unbound total ASA	UtASA
13	Unbound backbone ASA	UbASA
14	Unbound side-chain ASA	UsASA
15	Unbound polar ASA	UpASA
16	Unbound non-polar ASA	UnASA
17	Unbound total RASA	UtRASA
18	Unbound backbone RASA	UbRASA
19	Unbound side-chain RASA	UsRASA
20	Unbound polar RASA	UpRASA
21	Unbound non-polar RASA	UnRASA

22	Unbound total mean DI	UtmDI
23	Unbound side-chain mean DI	UsmDI
24	Unbound maximum DI	UmaxDI
25	Unbound minimal DI	UminDI
26	Unbound total mean PI	UtmPI
27	Unbound side-chain mean PI	UsmPI
28	Unbound maximum PI	UmaxPI
29	Unbound minimal PI	UminPI
30	Bound total ASA	BtASA
31	Bound backbone ASA	BbASA
32	Bound side-chain ASA	BsASA
33	Bound polar ASA	BpASA
34	Bound non-polar ASA	BnASA
35	Bound total RASA	BtRASA
36	Bound backbone RASA	BbRASA
37	Bound side-chain RASA	BsRASA
38	Bound polar RASA	BpRASA
39	Bound non-polar RASA	BnRASA
40	Bound total mean DI	BtmDI
41	Bound side-chain mean DI	BsmDI
42	Bound maximum DI	BmaxDI
43	Bound minimal DI	BminDI
44	Bound total mean PI	BtmPI
45	Bound side-chain mean PI	BsmPI
46	Bound maximum PI	BmaxPI
47	Bound minimal PI	BminPI
48	$(ASA_{unb}(total) - ASA_{bnd}(total))^{\frac{1}{2}}$	$DtASA^{\frac{1}{2}}$
49	$(ASA_{unb}(polar) - ASA_{bnd}(polar))^{\frac{1}{2}}$	$DpASA^{\frac{1}{2}}$
50	$(ASA_{unb}(non-polar) - ASA_{bnd}(non-polar))^{\frac{1}{2}}$	$DnASA^{\frac{1}{2}}$
51	$(RASA_{unb}(total) - RASA_{bnd}(total))^{\frac{1}{2}}$	$DtRASA^{\frac{1}{2}}$
52	$(RASA_{unb}(polar) - RASA_{bnd}(polar))^{\frac{1}{2}}$	$DpRASA^{\frac{1}{2}}$
53	$(RASA_{unb}(non-polar) - RASA_{bnd}(non-polar))^{\frac{1}{2}}$	$DnRASA^{\frac{1}{2}}$
54	$ASA_{unb}(total) - ASA_{bnd}(total)$	$DtASA$
55	$ASA_{unb}(polar) - ASA_{bnd}(polar)$	$DpASA$
56	$ASA_{unb}(non-polar) - ASA_{bnd}(non-polar)$	$DnASA$
57	$RASA_{unb}(total) - RASA_{bnd}(total)$	$DtRASA$
58	$RASA_{unb}(polar) - RASA_{bnd}(polar)$	$DpRASA$

59	$RASA_{unb}(non-polar)-RASA_{bnd}(non-polar)$	$DnRASA$
60	$\left(ASA_{unb}(total)-ASA_{bnd}(total)\right)^{\frac{3}{2}}$	$DtASA^{\frac{3}{2}}$
61	$\left(ASA_{unb}(polar)-ASA_{bnd}(polar)\right)^{\frac{3}{2}}$	$DpASA^{\frac{3}{2}}$
62	$\left(ASA_{unb}(non-polar)-ASA_{bnd}(non-polar)\right)^{\frac{3}{2}}$	$DnASA^{\frac{3}{2}}$
63	$\left(RASA_{unb}(total)-RASA_{bnd}(total)\right)^{\frac{3}{2}}$	$DtRASA^{\frac{3}{2}}$
64	$\left(RASA_{unb}(polar)-RASA_{bnd}(polar)\right)^{\frac{3}{2}}$	$DpRASA^{\frac{3}{2}}$
65	$\left(RASA_{unb}(non-polar)-RASA_{bnd}(non-polar)\right)^{\frac{3}{2}}$	$DnRASA^{\frac{3}{2}}$
66	$\left(ASA_{unb}(total)-ASA_{bnd}(total)\right)^{2}$	$DtASA^{2}$
67	$\left(ASA_{unb}(polar)-ASA_{bnd}(polar)\right)^{2}$	$DpASA^{2}$
68	$\left(ASA_{unb}(non-polar)-ASA_{bnd}(non-polar)\right)^{2}$	$DnASA^{2}$
69	$\left(RASA_{unb}(total)-RASA_{bnd}(total)\right)^{2}$	$DtRASA^{2}$
70	$\left(RASA_{unb}(polar)-RASA_{bnd}(polar)\right)^{2}$	$DpRASA^{2}$
71	$\left(RASA_{unb}(non-polar)-RASA_{bnd}(non-polar)\right)^{2}$	$DnRASA^{2}$
72	$\left(ASA_{unb}(total)-ASA_{bnd}(total)\right)^{\frac{5}{2}}$	$DtASA^{\frac{5}{2}}$
73	$\left(ASA_{unb}(polar)-ASA_{bnd}(polar)\right)^{\frac{5}{2}}$	$DpASA^{\frac{5}{2}}$
74	$\left(ASA_{unb}(non-polar)-ASA_{bnd}(non-polar)\right)^{\frac{5}{2}}$	$DnASA^{\frac{5}{2}}$
75	$\left(RASA_{unb}(total)-RASA_{bnd}(total)\right)^{\frac{5}{2}}$	$DtRASA^{\frac{5}{2}}$
76	$\left(RASA_{unb}(polar)-RASA_{bnd}(polar)\right)^{\frac{5}{2}}$	$DpRASA^{\frac{5}{2}}$
77	$\left(RASA_{unb}(non-polar)-RASA_{bnd}(non-polar)\right)^{\frac{5}{2}}$	$DnRASA^{\frac{5}{2}}$
78	Conservation	CNSV
79	Relative conservation of the actual residue compared to the alanine on a certain position	CNSV_REL1
80	Relative conservation of the residue with maximum percentage compared to the alanine on a certain position	CNSV_REL2
81	Logarithm of CNSV_REL1	logCNSV_REL1
82	Logarithm of CNSV_REL2	logCNSV_REL2

Table S5. The numerical values of 10 different kinds of properties of the 20 amino acids

Residue	Na [a]	Nec	Nphb	Hdrpo	Hdrpi	Prop	Isoep	Mass	Enc	Eiip
A	5	0	2	0.25	3	-0.17	6.11	71.1	-22	0.0373
C	6	0	2	0.04	-1	0.43	6.31	103.1	4.66	0.0829
D	8	-1	4	-0.72	3	-0.38	5.945	115.1	-4.12	0.1263
E	9	-1	4	-0.62	3	-0.13	5.785	129.1	-3.64	0.0058
F	11	0	2	0.61	-2.5	0.82	5.755	147.2	5.27	0.0946
G	4	0	2	0.16	0	-0.07	6.065	57	-1.62	0.005
H	10	0	4	-0.4	-0.5	0.41	5.565	137.1	1.28	0.0242
I	8	0	2	0.73	-1.8	0.44	6.04	113.2	5.58	0

K	9	1	2	-1.1	3	-0.36	5.61	128.2	-4.18	0.0371
L	8	0	2	0.53	-1.8	0.4	6.035	113.2	5.01	0
M	8	0	2	0.26	-1.3	0.66	5.705	131.2	3.51	0.0823
N	8	0	4	-0.64	0.2	0.12	5.43	114.1	-2.65	0.0036
P	7	0	2	-0.07	0	-0.25	6.295	97.1	-3.03	0.0198
Q	9	0	4	-0.69	0.2	-0.11	5.65	128.1	-2.76	0.0761
R	11	1	4	-1.76	-0.5	0.27	5.405	156.2	-0.93	0.0959
S	6	0	4	-0.26	0.3	-0.33	5.7	87.1	-2.84	0.0829
T	7	0	4	-0.18	-0.4	-0.18	5.595	101.1	-1.2	0.0941
V	7	0	2	0.54	-1.5	0.27	6.015	99.1	4.45	0.0057
W	14	0	3	0.37	-3.4	0.83	5.935	186.2	52	0.0548
Y	12	0	3	0.02	-2.3	0.66	5.705	163.2	2.15	0.0516

[a] The explanation of the 10 properties can be found in Table S4.

Table S6. Features selected from 82 features and the corresponding cross validation performance in SFS process

Round	Features identified	Accuracy	Recall	Precision	F-measure
1	BpRASA	0.760	0.790	0.671	0.726
2	BpRASA, DpASA	0.838	0.774	0.814	0.793
3	BpRASA, DpASA, $DnASA^{3/2}$	0.844	0.774	0.828	0.800
4	BpRASA, DpASA, $DnASA^{3/2}$, BnRASA	0.864	0.823	0.836	0.829
5	BpRASA, DpASA, $DnASA^{3/2}$, BnRASA, BbRASA	0.864	0.839	0.825	0.832
7	BpRASA, DpASA, $DnASA^{3/2}$, BnRASA, BbRASA, Enc	0.864	0.839	0.825	0.832

Table S7. The top 11 normalized features selected by decision tree, F-score and mRMR.

No.	decision tree [a]	F-score	mRMR
1	BsRASA(37)	BsRASA(37)	BtRASA(35)
2	UsASA (14)	BtRASA(35)	$DtASA^{1/2}$ (48)
3	$DpRASA^{1/2}$ (52)	BpRASA (38)	B factor (11)
4	BsmDI (41)	BsASA (32)	CNSV (78)
5	CNSV (78)	BsmPI (45)	Hdrpi (5)

6	UpRASA (20)	BtASA (30)	BminPI (47)
7	$CNSV\_REL1$ (79)	BtmPI (44)	$DnASA^{5/2}$ (74)
8	$DtASA^{1/2}$ (48)	BminPI (47)	BpRASA (38)
9	UpASA (15)	BpASA (33)	BtmDI (40)
10	UtmDI (22)	BnRASA (39)	BsASA (32)
11	Hdrpo (4)	BsmDI (41)	$DtASA^{3/2}$ (60)

[a] The numbers in the parentheses of columns 2-4 are the feature number in the Table S4.

Table S8. Features selected and the corresponding cross-validation performance in PSFS process for normalized features

Round	Features identified	Accuracy	Recall	Precision	F-measure
1	$CNSV\_REL1$, BsRASA	0.766	0.790	0.681	0.731
	$CNSV\_REL1$, BpRASA	0.766	0.710	0.710	0.710
	$CNSV\_REL1$, BtRASA	0.766	0.694	0.717	0.705
2	$CNSV\_REL1$, BsRASA, UpASA	0.805	0.790	0.742	0.766
	$CNSV\_REL1$, BtRASA, UpASA	0.799	0.807	0.725	0.763
	$CNSV\_REL1$, BsRASA, BtmDI	0.812	0.742	0.780	0.760
3	$CNSV\_REL1$, BsRASA, UpASA, BminPI	0.831	0.790	0.790	0.790
	$CNSV\_REL1$, BsRASA, BtmDI, UsASA	0.825	0.774	0.787	0.780
	$CNSV\_REL1$, BtRASA, UpASA, BpRASA	0.812	0.807	0.746	0.775
4	$CNSV\_REL1$, BsRASA, BtmDI, UsASA, BminPI	0.831	0.823	0.773	0.796
	$CNSV\_REL1$, BtRASA, UpASA, BpRASA, B factor	0.825	0.839	0.754	0.794

	$CNSV\_REL1$, BsRASA, UpASA, BminPI, $DpRASA^{1/2}$	0.825	0.823	0.761	0.791
5	$CNSV\_REL1$, BsRASA, BtmDI, UsASA, BminPI, UtmDI	0.838	0.823	0.785	0.803
	$CNSV\_REL1$, BtRASA, UpASA, BpRASA, B factor, BtmDI	0.831	0.855	0.757	0.803
	$CNSV\_REL1$, BtRASA, UpASA, BpRASA, B factor, BsmDI	0.831	0.855	0.757	0.803
6	$CNSV\_REL1$, BtRASA, UpASA, BpRASA, B factor, BtmDI, UtmDI	0.857	0.839	0.813	0.825
	$CNSV\_REL1$, BtRASA, UpASA, BpRASA, B factor, BtmDI, BsASA	0.838	0.871	0.761	0.812
	$CNSV\_REL1$, BsRASA, BtmDI, UsASA, BminPI, UtmDI, BsmDI	0.844	0.823	0.797	0.810
7	$CNSV\_REL1$, BtRASA, UpASA, BpRASA, B factor, BtmDI, UtmDI, $DpRASA^{1/2}$	0.844	0.823	0.797	0.810
	$CNSV\_REL1$, BtRASA, UpASA, BpRASA, B factor, BtmDI, UtmDI, BsRASA	0.838	0.823	0.785	0.803
	$CNSV\_REL1$, BtRASA, UpASA, BpRASA, B factor, BtmDI, UtmDI, $DtASA^{1/2}$	0.838	0.807	0.794	0.800

Table S9. Consensus results based on combining any two of the five models (MINERVA2, APIS, KFC2a, KFC2b, Our model).

Methods	Accuracy	Specificity	Recall	Precision	F-measure
Our-APIS	0.6631	0.5672	**0.8929**	0.4630	0.6098
Our-MIN	0.7053	0.6418	0.8571	0.5000	*0.6316*
Our-KFC2a	0.6737	0.5821	**0.8929**	0.4717	0.6173
Our-KFC2b	0.6947	0.6269	0.8571	0.4898	*0.6233*
APIS-KFC2b	0.7053	0.7164	0.6786	0.5000	*0.5758*
MIN-KFC2b	0.7263	**0.7910**	0.5714	0.5333	*0.5517*
KFC2a-KFC2b	**0.7368**	0.7164	0.7857	**0.5366**	**0.6377**
APIS-KFC2a	0.6947	0.6567	0.7857	0.4889	0.6027
MIN-KFC2a	0.7263	0.7015	0.7857	0.5238	0.6286
APIS-MIN	0.7263	0.7612	0.6429	0.5294	*0.5806*

Table S10. Interface information referred to the interfaces in the independent test set.

Interfaces	Recorded in SCOPPI?	SIZE	$\Delta ASA/(Å^2)$ [a]
1CDL(A:E)	No	Small	1394

1DVA(H:X)	No	Small	611
1DX5(N:J)	Yes	Small	874
1EBP(A:C)	Yes	Small	521
1EBP(A:D)	Yes	Small	314
1ES7(A:B)	Yes	Medium	1679
1ES7(A:D)	Yes	Small	777
1FAK(T:H)	Yes	Small	1147
1FAK(T:L)	Yes	Small	753
1FAK(T:L)	Yes	Small	801
1FOE(A:B)	Yes	Large	3056
1G3I(A:G)	Yes	Small	1055
1G3I(A:H)	Yes	Small	699
1GL4(A:B)	Yes	Large	2007
1IHB(A:B)	Yes	Small	659
1JAT(A:B)	Yes	Medium	1468
1JPP(B:D)	No	Small	551
1MQ8(A:B)	Yes	Small	1241
1NUN(A:B)	Yes	Large	2086
1UB4(A:C)	Yes	Large	2785
2HHB(A:B)	Yes	Medium	1649

[a] The ΔASA of 1CDL,1DVA, 1JPP were calculated by NACCESS [1], because the interfaces were not recorded in SCOPPI [2].

Table S11. Statistical performance of our model for predicting hotspots of the independent test set by the types of protein-protein interfaces.

Types of interfaces	Accuracy	Specificity	Recall	Precision	F-measure
All residues (28 HS/ 67 NS)	0.705	0.657	0.821	0.500	0.621
SCOPPI interface (24 HS/ 49 NS)	0.753	0.657	0.833	0.588	**0.689**
Non SCOPPI interface (4 HS/18 NS)	0.545	0.714	0.75	0.250	0.375
Small interface (19 HS/56 NS)	0.707	0.500	0.789	0.455	0.577
Medium interface (3 HS/4 NS)	0.714	0.679	1.00	0.6	**0.750**
Large interface (6 HS/7 NS)	0.692	0.500	0.833	0.625	**0.714**

Table S12. The top 10 features selected by decision tree, F-score, and mRMR from the old 48 features reported in Xia et al.

No.	Decision tree [a]	F-score	mRMR
1	BsRASA (37)	BsRASA (37)	BtRASA (35)
2	UsASA (14)	BtRASA (35)	B factor (11)
3	CNSV (78)	BpRASA (38)	CNSV(78)
4	BsmDI (41)	BsASA (32)	Hdrpi (5)
5	UmaxPI (28)	BsmPI (45)	BminPI (47)
6	UpASA (15)	BtASA (30)	BpRASA (38)
7	Prop (6)	BtmPI (44)	BtmDI (40)
8	UtmDI (22)	BminPI (47)	UminDI (25)
9	UnASA (16)	BpASA (33)	BnRASA (39)
10	Eiip (10)	BnRASA (39)	BsASA (32)

[a] The numbers in the parentheses of columns 2-4 are the feature number in the Table S4.

Table S13. Features selected and the corresponding cross-validation performances in PSFS process for the 48 old features reported in Xia et al.'s paper [3].

Round	Features identified	Accuracy	Recall	Precision	F-measure
1	BsRASA	0.760	0.790	0.671	0.726
	BpRASA	0.714	0.823	0.607	0.699
	BtRASA	0.753	0.710	0.688	0.698
2	BsRASA, UsASA	0.818	0.774	0.774	0.774
	BsRASA, BsASA	0.812	0.790	0.754	0.772
	BsRASA, UpASA	0.773	0.839	0.675	0.748
3	BsRASA, UsASA, BsASA	0.851	0.774	0.842	0.807
	BsRASA, UsASA, UnASA	0.838	0.807	0.794	0.800
	BsRASA, UpASA, BpRASA	0.812	0.855	0.726	0.785

	BsRASA, UsASA, UnASA, UpASA	0.851	0.807	0.820	0.813
4	BsRASA, UsASA, UnASA, B factor	0.851	0.807	0.820	0.813
	BsRASA, UsASA, UnASA, BtmDI	0.844	0.823	0.797	0.810
	BsRASA, UsASA, UnASA, B factor, CNSV	0.864	0.807	0.848	0.826
5	BsRASA, UsASA, UnASA, BtmDI, BsmDI	0.857	0.823	0.823	0.823
	BsRASA, UsASA, UnASA, B factor, BsmDI	0.857	0.823	0.823	0.823
	BsRASA, UsASA, UnASA, B factor, CNSV, UtmDI	0.870	0.823	0.850	0.836
6	BsRASA, UsASA, UnASA, B factor, CNSV, UmaxPI	0.864	0.807	0.848	0.826
	BsRASA, UsASA, UnASA, B factor, CNSV, UminDI	0.864	0.807	0.848	0.826
	BsRASA, UsASA, UnASA, B factor, CNSV, UtmDI, UminDI	0.870	0.823	0.850	0.836
7	BsRASA, UsASA, UnASA, B factor, CNSV, UtmDI, BminPI	0.870	0.823	0.850	0.836
	BsRASA, UsASA, UnASA, B factor, CNSV, UmaxPI, UtmDI	0.864	0.823	0.836	0.829

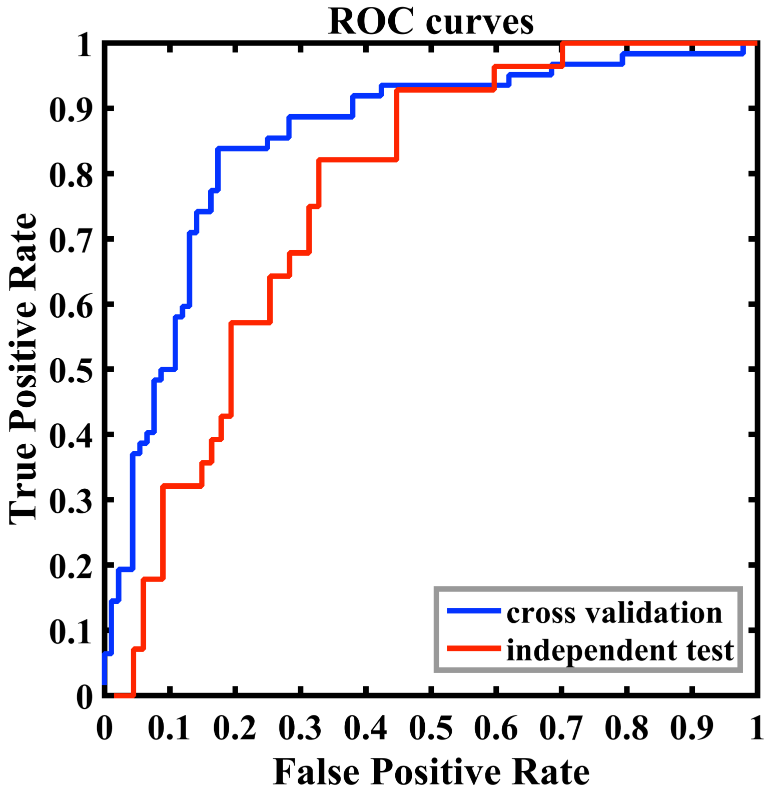

Figure S1. The ROC curves for cross-validation results of the training data set and the predictive results of the independent test set.

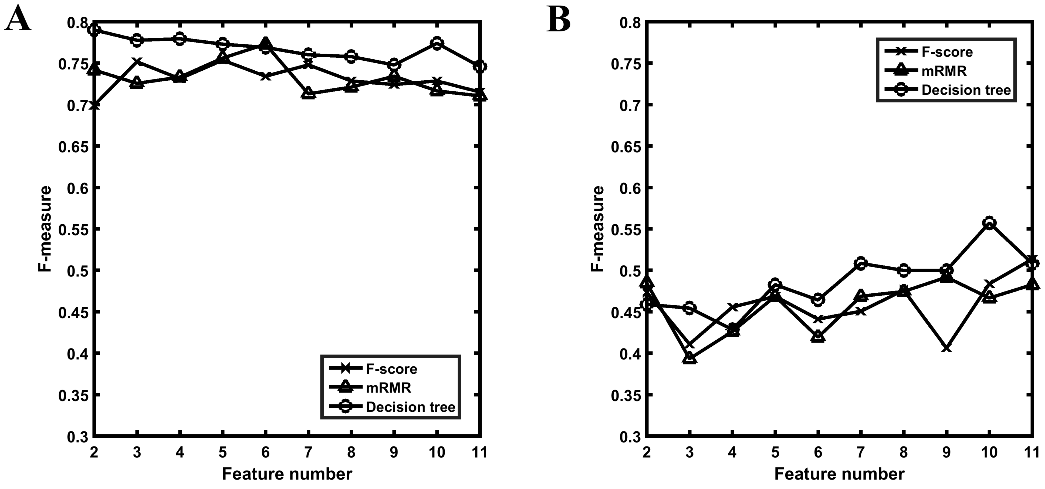

Figure S2. The F-measures based on different number of normalized features selected by different methods. A. F-measures on the cross validation; B. F-measures on the independent test set.

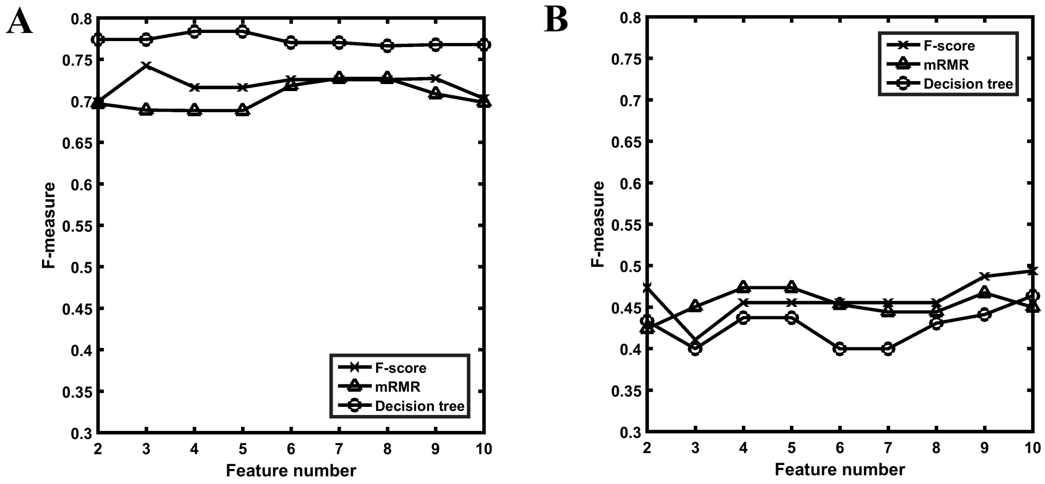

Figure S3. The F-measures based on different number of features selected by different methods from the 48 old features selected by different methods. A. F-measures on the cross validation; B. F-measures on the independent test set.

1. Hubbard SJ, Thornton JM: **Naccess. Computer Program, Department of Biochemistry and Molecular Biology**. *University College London* 1993, **2**(1).
2. Winter C, Henschel A, Kim WK, Schroeder M: **SCOPPI: a structural classification of protein-protein interfaces**. *Nucleic acids research* 2006, **34**(Database issue):D310-314.

3. Xia JF, Zhao XM, Song J, Huang DS: **APIS: accurate prediction of hot spots in protein interfaces by combining protrusion index with solvent accessibility**. *BMC bioinformatics* 2010, **11**:174.
